# A gender specific risk assessment of coronary heart disease based on physical examination data

**DOI:** 10.1038/s41746-023-00887-8

**Published:** 2023-07-31

**Authors:** Hui Yang, Ya-Mei Luo, Cai-Yi Ma, Tian-Yu Zhang, Tao Zhou, Xiao-Lei Ren, Xiao-Lin He, Ke-Jun Deng, Dan Yan, Hua Tang, Hao Lin

**Affiliations:** 1 School of Computer Science, Chengdu University of Information Technology, Chengdu, 610225 China; 2 School of Life Science and Technology, Center for Informational Biology, University of Electronic Science and Technology of China, Chengdu, 610054 China; 3 School of Medical Information and Engineering, Southwest Medical University, Luzhou, 646000 China; 4 Medical Engineering & Medical Informatics Integration and Transformational Medicine Key Laboratory of Luzhou City, Luzhou, 646000 China; 5 Sichuan Chuanjiang Science and Technology Research Institute Co., Ltd, Luzhou, 646000 China; 6 Beijing Institute of Clinical Pharmacy, Beijing Friendship Hospital, Capital Medical University, Beijing, 100050 China; 7 School of Basic Medical Sciences, Southwest Medical University, Luzhou, 646000 China; 8 Central Nervous System Drug Key Laboratory of Sichuan Province, Luzhou, 646000 China

**Keywords:** Risk factors, Physical examination

## Abstract

Large-scale screening for the risk of coronary heart disease (CHD) is crucial for its prevention and management. Physical examination data has the advantages of wide coverage, large capacity, and easy collection. Therefore, here we report a gender-specific cascading system for risk assessment of CHD based on physical examination data. The dataset consists of 39,538 CHD patients and 640,465 healthy individuals from the Luzhou Health Commission in Sichuan, China. Fifty physical examination characteristics were considered, and after feature screening, ten risk factors were identified. To facilitate large-scale CHD risk screening, a CHD risk model was developed using a fully connected network (FCN). For males, the model achieves AUCs of 0.8671 and 0.8659, respectively on the independent test set and the external validation set. For females, the AUCs of the model are 0.8991 and 0.9006, respectively on the independent test set and the external validation set. Furthermore, to enhance the convenience and flexibility of the model in clinical and real-life scenarios, we established a CHD risk scorecard base on logistic regression (LR). The results show that, for both males and females, the AUCs of the scorecard on the independent test set and the external verification set are only slightly lower (<0.05) than those of the corresponding prediction model, indicating that the scorecard construction does not result in a significant loss of information. To promote CHD personal lifestyle management, an online CHD risk assessment system has been established, which can be freely accessed at http://lin-group.cn/server/CHD/index.html.

## Introduction

Coronary angiography is the gold standard for the diagnosis of coronary heart disease (CHD). However, due to economic constraints or fear of invasive examination, many patients miss the opportunity of early diagnosis and treatment^1^. Therefore, it is crucial to explore new methods for large-scale noninvasive or minimally invasive screening for CHD risk. Physical examination is an important way to detect diseases early. Therefore, the development of CHD risk assessment tools based on physical examination data is significant to conduct large-scale disease screening.

Risk assessment tools for diseases have been established by quantifying the risk factors associated with the disease^2–5^. Internationally renowned coronary risk assessment tools include: Framingham Risk Score (FRS)^6^, Global Registry of Acute Coronary Event (GRACE)^7^, Systematic Coronary Risk Estimation (SCORE) and China-PAR 10-year risk prediction model^3,8^. These risk assessment tools played an indispensable role in the prevention of CHD. In 1961, Framingham first proposed the concept of risk factors for CHD, which became the cornerstone of cardiovascular disease epidemiological research thereafter. The Framingham Heart Study (FHS) subsequently reported the effects of various risk factors, such as age, blood pressure, cholesterol, smoking, and obesity. The GRACE score is a predictive tool used to assess the risk of death or recurrent myocardial infarction during hospitalization and after discharge in patients with acute coronary syndrome (ACS). It is a valuable basis for helping physicians choose early treatment strategies. The SCORE scoring system incorporates data from the European Systematic Coronary Risk Evaluation project, which included populations from 12 European countries and assessed the 10-year risk of fatal cardiovascular disease. In the SCORE scoring system, the risk factors include age, gender, total cholesterol level, systolic blood pressure, and smoking. The China-PAR score takes into account the differences between Chinese and Western populations in disease spectrum and the prevalence of cardiovascular disease risk factors. Therefore, the China-PAR scoring system is suitable for the Chinese population. The risk factors used in the system include gender, age, place of residence (urban or rural), region (north or south, with the Yangtze River as the boundary), waist circumference, total cholesterol, high-density lipoprotein cholesterol, current blood pressure level, use of antihypertensive medication, presence of diabetes, current smoking status, and family history of cardiovascular disease. Although the risk factors involved in these studies were obtained through cohort studies, which provided a good foundation for CHD risk assessment, cohort studies may not simultaneously investigate a large number of or special risk factors to avoid introducing too many confounding factors. In addition, the application scenarios of these risk assessment tools are not explicitly stated for large-scale disease screening.

Therefore, we aim to establish a CHD risk assessment tool based on physical examination data, specifically tailored to the application scenario of physical examinations. The physical examination system facilitates the storage and management of physical examination data, and the establishment of a risk assessment model using deep learning can effectively screen the risk of a large number of individuals within the physical examination system. Additionally, a 100-point score card can also be established to allow individuals to assess their own disease risk and manage risk factors conveniently.

**Fig. 1:**
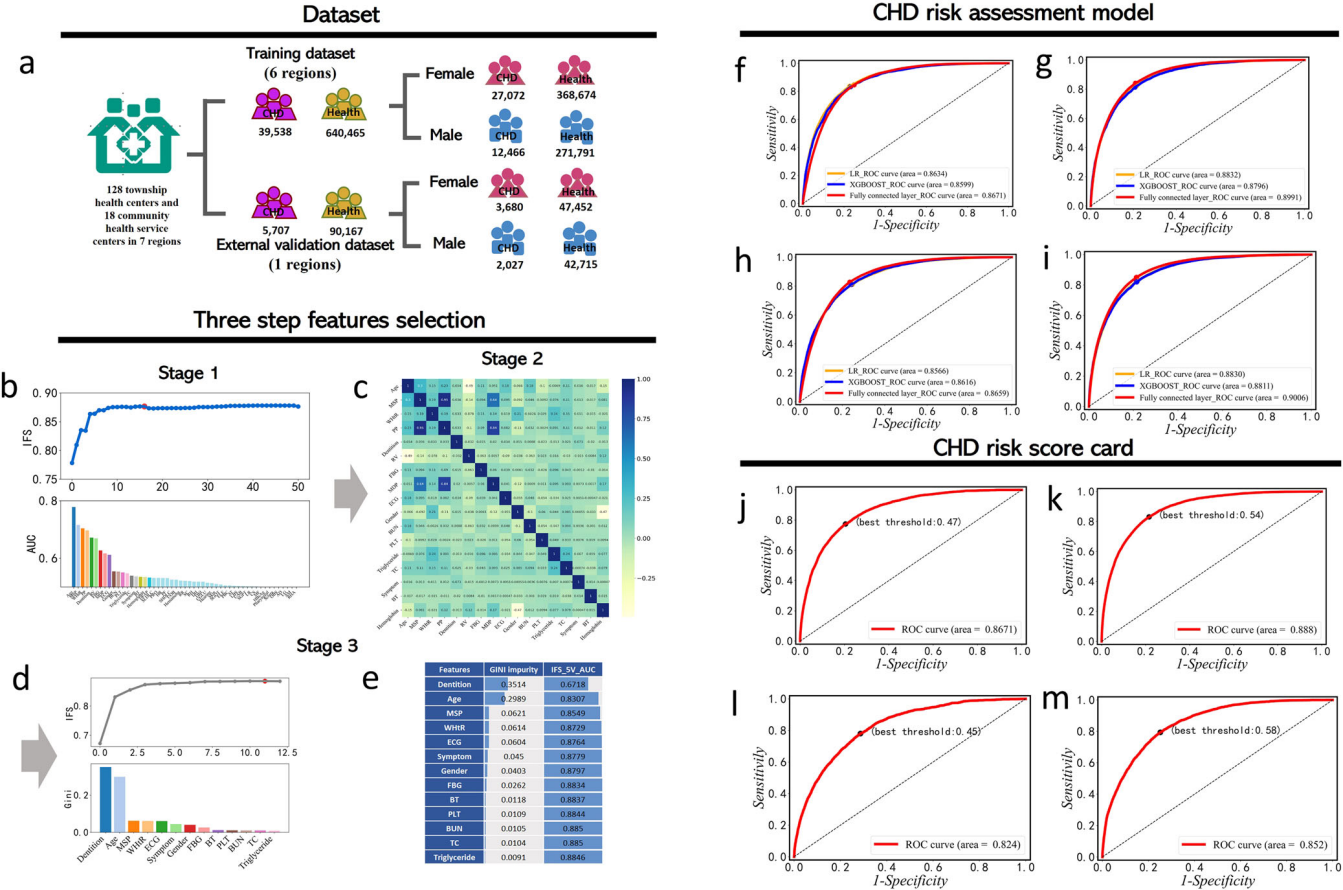
The flow chart to show the benchmark data, feature selection, and prediction performance of CHD risk assessment. **a** gender-specific training dataset and external dataset based on physical examination data. Among them, purple indicates patients with CHD regardless of gender, and yellow indicates healthy people regardless of gender. The pink shows the female population and the blue shows the male population. The three-step feature screening scheme is as follows: **b** Stage 1 is an IFS curve (blue curve) generated based on the AUC sorting of a single feature. The red dot of the blue curve represents the maximum AUC. The different colors of the bar plot represent different features in the feature subset, and the height of the bar plot represents the AUC value of a single feature. **c** Stage 2 is a heat map used to show Pearson correlations between pairwise features. **d** Stage 3 refers to the IFS curve (gray curve) generated after feature sorting based on the Gini coefficient. Red points on the gray curve represent the maximum value of AUC. Different colors of the histogram represent different features, and the height of the histogram represents the Gini coefficient value of a single feature. **e** The Gini impurity of each feature in Stage 3 and the AUC values of feature subsets generated by IFS strategy. **f** The ROC curves of CHD risk assessment model on male training cohort. **g** The ROC curves of CHD risk assessment model on female training cohort. **h** The ROC curves of the CHD risk assessment model on external validation cohort of males. **i** The ROC curves of the CHD risk assessment model on an external validation cohort of females. **j** The ROC curves of CHD risk scorecard on training cohort of males. **k** The ROC curves of CHD risk scorecard on training cohort of females. **l** The ROC curves of CHD risk scorecard on external validation cohort of males. **m** The ROC curves of CHD risk scorecard on external validation cohort of females.

This study is a retrospective multi-center case-control study. Initially, we preprocess the data and select a group of non-redundant and highly informative CHD risk factors through a three-step feature screening scheme based on physical examination indicators. Subsequently, we construct a gender-specific CHD risk assessment model using deep learning techniques. Moreover, we employ a logistic regression (LR) model and a scorecard scaling algorithm to develop a gender-specific CHD risk scorecard. The flowchart is illustrated in Fig. 1. Furthermore, this study collected questionnaire data on the lifestyles of CHD patients and healthy individuals from the same source. By comparing the lifestyle differences between CHD patients and healthy individuals, we provide insights into health management and intervention programs for CHD patients. To facilitate clinical use, the corresponding webserver calculation tool was developed in this study, which can calculate the risk of CHD in individuals online. The CHD Risk assessment tool (named CHD Risk Score Card) can be freely accessed from http://lin-group.cn/server/CHD/index.html.

## Results

### The preparation of physical examination data

A sufficient amount of real data is the basis for constructing a reliable high-performance model. We obtained the physical examination data from January 2018 to October 2021 from the Electronic Medical Record (EMR) of the Luzhou Municipal Health Commission in China. The sample includes healthy individuals and CHD patients diagnosed with luminal stenosis with a diameter ≥50% through coronary angiograph.

After preprocessing of the samples and physical examination indicators, we acquired a dataset of 39,538 CHD patients and 640,465 healthy individuals. The average age (standard deviation (SD)) of CHD patients is 70.95 (8.51) years, of which 31.53% are male and 68.47% are female. The average age (SD) of healthy people is 58.50 (13.71) years, of which 42.44% are male and 57.56% are female. The dataset was randomly divided into a training set for model construction and a test set for internal verification in a ratio of 7:3 (Fig. 1a). Furthermore, we also obtained external validation cohort data, including 5,707 CHD patients and 90,167 healthy people. The mean age (SD) of CHD patients is 69.93 (8.49) years, with males accounting for 35.52% and females accounting for 64.48%. The mean age (SD) of healthy people is 59.48 (13.66) years, with 47.37% males and 52.63% females (Supplementary Table 1).

After preprocessing the questionnaire survey on the lifestyle habits of CHD patients and healthy individuals, a total of 45,242 CHD patients and 730,348 healthy individuals were collected (Supplementary Table 2). The questionnaire data reflects the lifestyle habits of CHD patients after the diagnosis of CHD. Additionally, after preprocessing the questionnaire survey on comorbidities of CHD and healthy people, there were 47,947 samples from CHD patients and 780,938 samples from healthy individuals (Supplementary Table 3).

The physical examination data included the following characteristics: age, gender, waist-to-height ratio (WHtR), mean systolic pressure (MSP), pulse pressure (PP), mean diastolic pressure (MDP), symptoms, body temperature (BT), heart rate (HR), pulse frequency (PF), respiratory rate (RR), dorsal foot artery pulsates (DFAP), rhythm of the heart (RH), lung breath sounds (LBS), cardiac souffle (CS), lung rale (LR), dentition, vision, hearing, skin, edema of lower extremity (ELE), pulmonary barrel chest (PBC), abdominal tenderness (AT), motor function (MF), pharyngeal, sclera, anus dre (AD), lymph gland (LG), abdominal mass (AM), fasting blood glucose (FBG), electrocardiograph (ECG), blood urea nitrogen (BUN), platelet count (PLT), triglyceride, total cholesterol (TC), hemoglobin, serum low-density lipoprotein cholesterol (LDL), serum high-density lipoprotein cholesterol (HDL), urine occult blood (UOB), hemameba, serum creatinine (SC), total bilirubin (TBil), urine glucose (UGLU), serum glutamic oxalacetic transaminase (SGOT), urine acetone bodies (UAB), serum glutamic pyruvic transaminase (SGPT), hepatitis B surface antigen (HBsAg), occult blood in stool (OBS), and RH antibody (RHA).

These features encompass both continuous variables and discrete variables. For continuous variables, we calculated the average value and variance of the feature, while for discrete variables, we used the label coding method to encode them and calculated the frequency of each discrete value in the feature (Supplementary Table 1).

### Selection of CHD risk factors

To identify representative and stable features from the initial physical examination indicators, we conducted a three-stage feature screening for 50 features.

In the first stage, logistic regression (LR) was utilized as the base classifier to evaluate the classification capability of each feature using 5-fold cross-validation. The features were then ranked based on their AUCs. By employing Incremental Feature Selection (IFS), 17 relevant indicators were filtered out (Fig. 1b, Supplementary Table 4).

In the second stage, the correlation between 17 features was observed using Pearson correlation coefficient (PCC) (Fig. 1c). If the correlation coefficient between the two features exceeded 0.4, we retained the feature with the higher AUC and removed the one with the lower AUC. For instance, features such as mean diastolic pressure (MDP), pulse pressure (PP), hemameba, and right ventricle (RV) were eliminated due to their correlation with other features (MDP and PP, Hemameba and Gender, RV and Age) exceeding the threshold of 0.4.

The third stage involved a comprehensive ranking of the remaining features using the Gini impurity method. We combined this ranking with IFS to select the final feature subset (Fig. 1d, e). This optimal feature subset consists of 11 features (Supplementary Table 5), including demographics (Age, Gender, WHtR), vital signs (MSP, Symptom, BT), external checkup (Dentition) and laboratory data (ECG, FBG, PLT, BUN).

The three-stage feature screening scheme ensures transparency throughout the process, resulting in selected features that are easier to interpret and comprehend, while also yielding stable results. The first stage quantifies the contribution of each individual feature to CHD. In the second stage, we assess redundancy between features using the intuitive and straightforward PCC. The third stage employs a model-based feature selection scheme to enhance the model’s performance. Furthermore, it is worth noting that both this study and some published works have found gender differences in CHD patients. Therefore, we have established separate datasets for males and females. The detailed datasets division has been shown in Supplementary Table 6 and Supplementary Table 7.

### CHD risk assessment model

We inputted the 10 optimal features (Age, WHtR, MSP, Symptom, BT, Dentition, ECG, FBG, PLT, BUN) into three different algorithms, namely Fully Connected Network (FCN), Logistic Regression (LR), and eXtreme Gradient Boosting (XGBoost) to build gender specific CHD risk assessment models and compare their performance on the internal validation set. The evaluation metrics including AUC, accuracy, precision, recall, and F1 score for the three models were recorded in Table 1 and depicted in Fig. 1f–i. It is evident that the performance of the three algorithms is quite similar, with the FCN model slightly outperforming the other two models, with AUCs of 0.8671 for males and 0.8991 for females. On the external validation set, as shown in Table 1 and Fig. 1h, i, the FCN model exhibits consistent and stable performance, with AUCs of 0.8659 and 0.9006 for males and females, respectively. When the data is straightforward and features are linearly independent, the fitting results of various algorithms tend to be similar. However, in the current dataset, the FCN model exhibits slightly better performance, which may be due to its ability to capture and learn nonlinear features in the intermediate layers.

**Table 1:** Performance metrics of the CHD risk assessment model.

		Male					Female				
	Algorithm	Recall	Accuracy	Precision	F1	AUC	Recall	Accuracy	Precision	F1	AUC
Training dataset	LR	0.6177	0.8743	0.3127	0.4152	0.8634	0.7596	0.8315	0.6845	0.7201	0.8832
	XGBoost	0.6024	0.8742	0.5535	0.5769	0.8599	0.7578	0.8318	0.8285	0.7915	0.8796
	FCN	0.8494	0.7971	0.7739	0.8218	0.8671	0.8657	0.8271	0.8036	0.8335	0.8991
External verification dataset	LR	0.6060	0.8743	0.3177	0.4169	0.8616	0.7571	0.8306	0.6741	0.7132	0.8830
	XGBoost	0.6624	0.8377	0.5010	0.5705	0.8621	0.8083	0.7941	0.7336	0.7691	0.8811
	FCN	0.8394	0.7970	0.7737	0.8052	0.8659	0.8663	0.8282	0.8050	0.8345	0.9006

**Fig. 2:**
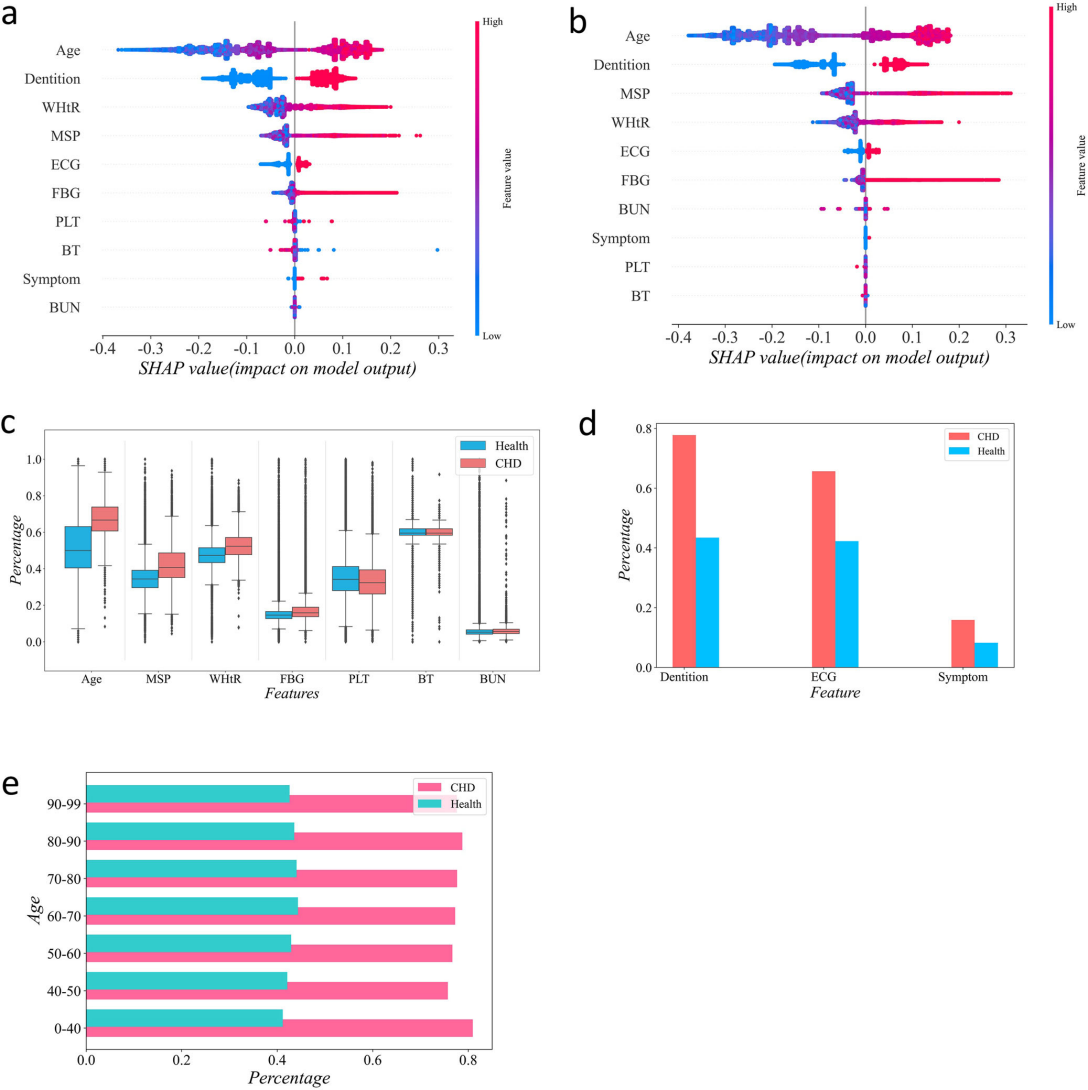
Statistical results of risk factors for CHD. **a** SHAP summary plot for male CHD risk model. **b** SHAP summary plot for female CHD risk model. **c** Statistical analysis for the seven optimal continuous features in healthy people (blue) and CHD patients (red). **d** Statistical analysis for the three optimal discrete features in healthy people (blue) and CHD patients (red). **e** The proportion of abnormal dentition in healthy people (blue) and CHD patients (blue) in different age groups.

To understand the contribution of features to the model, we conducted an interpretability analysis using SHAP (SHapley Additive exPlanations). Figure 2 displays the SHAP plot representing all sample points, with color indicating the magnitude of the feature values (red for large values, blue for small values, and purple for values near the mean). The magnitude of the values on the *x*-axis represents their impact on the model. From Fig. 2a and Fig. 2b, it is evident that the younger the age, the lower the risk, while the older the age, the higher the risk. However, when age increases to a certain range, the impact on the model is limited. When WHtR, MSP, and FBG values are below or equal to the mean, their impact on the risk of CHD is minimal. However, when these values exceed the mean, their influence on the risk of CHD significantly increases as the values increase. Additionally, we also performed statistical analysis on the seven optimal continuous features (Fig. 2c), three optimal discrete features (Fig. 2d) in healthy individuals and CHD patients, as well as the proportion of abnormal dentition (Fig. 2e) in healthy individuals and CHD patients of different age groups.

### CHD risk scorecard

To improve the convenience of clinical application, we developed a CHD risk scorecard using the same set of features as the CHD risk assessment model. In the scorecard, we first discretized the continuous variables by binning, allowing users to assign scores based on their respective indicators. Subsequently, by calculating the Weight of Evidence (WoE) value of each bin and mapping these values back to the datasets, we built a logistic regression (LR) model.

On the internal validation set, the LR-based model could produce the AUCs of 0.8668 and 0.8884 for male and female samples, respectively. Similarly, on the external validation set, the AUCs for male and female samples were 0.8238 and 0.8519, respectively (Table 2, and Fig. 1j–m). Compared with the CHD risk assessment model, this model shows a relatively small performance loss, indicating that the binning process has minimal impact on the model. Thus, the chosen binning method has been proven to be reasonable.

**Table 2:** Performance metrics of the CHD risk scorecard using LR.

	Male					Female				
	Recall	Accuracy	Precision	F1	AUC	Recall	Accuracy	Precision	F1	AUC
Training dataset	0.7458	0.8144	0.6781	0.7103	0.8668	0.8509	0.7667	0.7157	0.7774	0.8884
External verification dataset	0.7296	0.7531	0.5330	0.6160	0.8238	0.8370	0.7090	0.6169	0.7103	0.8519

**Fig. 3:**
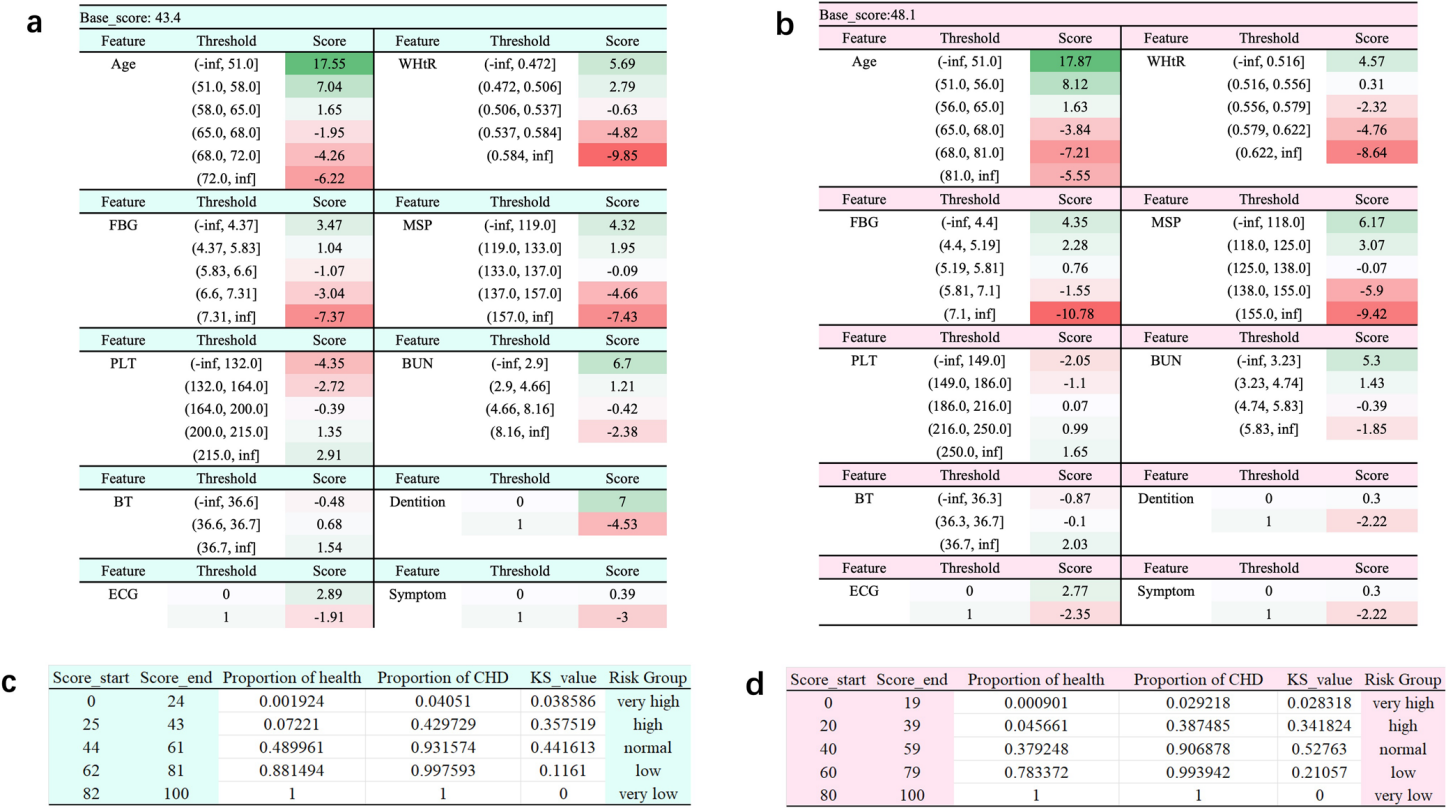
Gender-specific CHD risk scorecard and its threshold of risk group. If the symptoms included palpitation, chest tightness, dizziness, and headache, choose 1, otherwise choose 0; If there were caries, missing teeth, or dentures in the dentition, choose 1, otherwise choose 0;1 was selected when ECG was abnormal, and 0 was selected otherwise. **a** CHD risk scorecard for males. **b** CHD risk scorecard for females. **c** Threshold of risk group in the scorecard for male. **d** Threshold of risk group in the scorecard for females.

After modeling, the LR-based model was transformed into a percentage scorecard using a scoring algorithm. The scorecard consists of a basic score and individual bin scores for each feature (Fig. 3a, b). When utilizing the scorecard, the basic score was added to the score corresponding to the feature’s bin to obtain the total score. To determine risk intervals, we employed the Kolmogorov-Smirnov (KS) curve to describe the overall score distribution (Fig. 3c, d, Supplementary Fig. 1). The KS curve illustrates the changes in the sample proportion of CHD patients (green line), the proportion of healthy individuals (blue line), and the trend of difference between CHD and healthy people (red line) with a score from 0 to 100. From the KS curve, it can be seen that the proportion of CHD in the low score group accumulates faster, while the proportion of healthy people in the high score group accumulates faster. The red curve shows the variation process of the difference between CHD and healthy people at each score point. At the highest point of the red curve, the ability to distinguish between CHD and healthy people is the strongest. Therefore, the score corresponding to this point was set as the inflection point used to distinguish risk. The inflection points of the CHD risk scorecard for males and females are 61 and 59, respectively. In order to provide users with a more direct scoring effect, we divided five risk intervals according to the KS curve, namely, high, relatively high, medium, relatively low, and low risk levels.

We also provided an online CHD risk scoring tool that can be freely accessed via http://lin-group.cn/server/CHD/index.html. This tool enables individuals to calculate their CHD risk based on the ten indicators submitted online, thereby promoting personalized health management.

## Discussion

In this work, we design a cascading screening system for CHD risk evaluation based on physical examination data. This system includes a CHD risk assessment model for large-scale population screening in the physical examination system and a CHD risk scorecard for individual risk assessment and management. The application of the CHD risk assessment model is not only applicable to public health, but also facilitates the integration of wearable devices or smart home IoT systems, thereby providing early warning and continuous monitoring of individual risks.

Physical examination data has several advantages of easy access, wide coverage, and comprehensive indicators, making it well-suited for large-scale disease screening. We screened a set of features to predict the risk of CHD. Compared with a single indicator, the comprehensive indicators can improve the robustness, reliability, and accuracy of disease prediction. We visualized the distribution of the optimal features (Age, Gender, WHtR, MSP, Symptom, BT, Dentition, ECG, FBG, PLT, BUN) and emphasized the differences between patients with CHD and healthy individuals (Fig. 2c, d). Numerous studies have demonstrated a strong association between these characteristics and CHD. Among them, age is a prominent risk factor. The risk of CHD increases with age. Although men and women share similar risk factors, gender differences influence the pathophysiology of these indicators, for example, women often show atypical symptoms, leading to missed diagnoses of CHD^7,8^. In addition, dentition is closely related to the occurrence of CHD^9^. Studies have shown that *Porphyromonas gingivalis*, which can cause oral infections, can also invade the endothelial cells of human coronary arteries. Moreover, periodontitis can induce systemic inflammatory responses, and the activation of these inflammatory responses can lead to the instability of coronary plaque, thus leading to the onset of acute coronary syndrome. Through Fig. 2e, we found that the dentition status at all ages is significantly related to the incidence of CHD. More than 70% of CHD patients have missing teeth, caries, and dentures, while only about 40% of healthy people have missing teeth, caries, and dentures. WHtR is a lifestyle-related indicator that can be controlled and improved by controlling dietary structure and exercise habits, thereby reducing the risk of CHD^10^. Coronary artery stenosis can cause a variety of symptoms. From the initial 23 recorded symptoms, we selected the four most relevant symptoms of CHD, namely, palpitation, chest tightness, dizziness, and headache (Supplementary Table 8). Platelet activation is related to thrombosis. During the occurrence of CHD, due to the formation of plaques or thrombosis, a large amount of platelets need to be consumed, which may lead to the gradual reduction of PLT consumption^11^. Fasting blood glucose and blood urea nitrogen indicate that CHD is closely related to diabetes and kidney disease^12,13^.

Compared with other previous tools for CHD risk assessment, this study focuses on more risk factors and constructed a better performance model. For example, Framingham 10-year heart disease risk score focuses on risk factors such as age, high-density lipoprotein, systolic blood pressure, total cholesterol, and smoking status. The AUCs are 0.705 and 0.742, respectively for male and female^6^. China-PAR 10-year risk prediction model focuses on risk factors, such as gender, age, current residence (urban or rural), region (northern or southern), waist circumference, total cholesterol, high-density lipoprotein, blood pressure, use of antihypertensive medication, diabetes status, smoking status, and family history of cardiovascular disease. The C indexes of the model are 0.794 for men and 0.811 for women, respectively^3^. Our study includes not only important CHD risk factors, such as age, gender, blood pressure, and obesity-related factors, but also significant characteristics, such as dental status, symptoms, platelet count, blood urea nitrogen, and bleeding time. The addition of these features not only improves the performance of the CHD risk assessment tool, but also provides new feature dimensions for the health management of CHD.

**Fig. 4:**
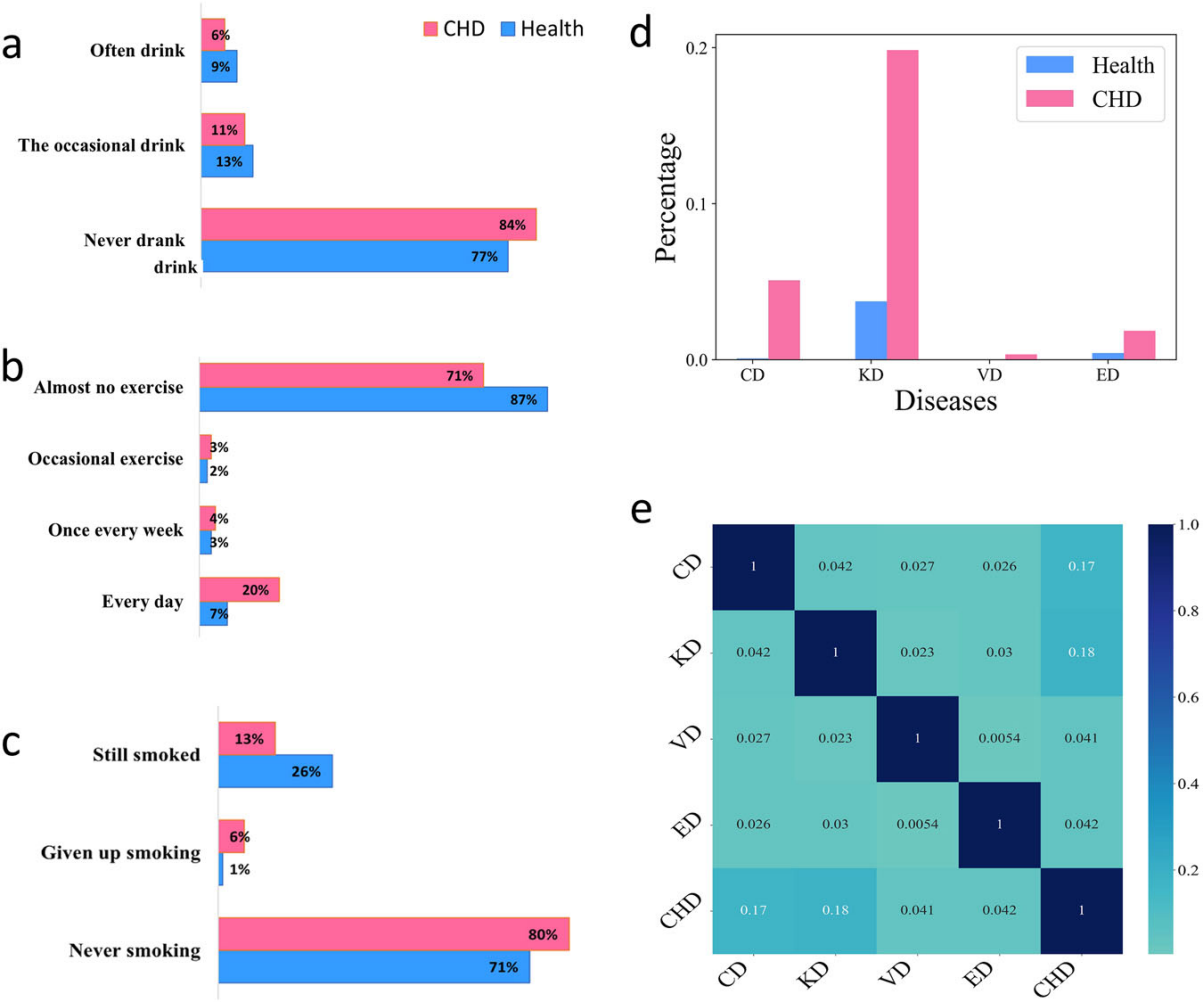
Living habits and comorbidities factors of CHD. **a** The proportion of drinking frequency in CHD patients (red) and healthy people (blue). **b** The proportion of exercise frequency in CHD patients (red) and healthy people (blue). **c** The proportion of smoking status in CHD patients (red) and healthy people (blue). **d** The proportion of comorbidity in CHD patients and healthy people. **e** Heat map of comorbidities.

Lifestyle is considered to be the simplest intervention way in the field of public health, and therefore attracts much attention. In this study, the lifestyle habits obtained through a questionnaire survey include smoking, drinking frequency, and exercise frequency. By comparing the lifestyle of CHD patients and healthy people, we can observe the implementation of the intervention on the above life habits of CHD patients (Fig. 4). According to observations, the frequency of alcohol consumption in patients with coronary heart disease is usually lower than that of healthy individuals (Fig. 4a). And The exercise frequency of patients with coronary heart disease is higher than that of healthy individuals (Fig. 4b). From Fig. 4c, we notice that the proportion of smoking cessation in CHD patients is much higher than that in healthy people. The above findings indicate that patients with CHD consciously improve their lifestyle and manage their living habits to prevent diseases after understanding their own physical condition^14–16^.

Comorbidity analysis plays a significant role in clinical decision-making, prognosis, and disease management. Figure 4d shows that the proportion of CHD patients with cerebrovascular disease (CD), kidney disease (KD), vascular disease (VD), and eye disease (ED) is higher than that of healthy people. Figure 4e shows that cerebrovascular disease has the strongest correlation with CHD (0.19), followed by kidney disease (0.18). This fully indicates that CHD patients are more likely to contract other diseases than healthy individuals.

The CHD risk assessment system designed in this study can be combined with China’s Nationwide Basic Public Health Services Project, which focuses on providing health management services for all residents, including free physical examinations^17^. However, it should be noted that the data sources of the current model are relatively limited in terms of geographical region, which hampers the examination of performance differences across regions. Future research should collect data from diverse regions to test the predictive accuracy of the evaluation system, analyze the impact of regional differences on CHD risk factors, and establish a more precise prediction system. Additionally, collecting ECG data will contribute to a more in-depth study of the ECG differences between CHD and other heart diseases. Furthermore, combining various heterogeneous data types with different deep learning methods can enhance the predictive performance of the model. It is also crucial to regularly monitor and evaluate the system’s discriminative ability, stability, and make necessary modifications as more data becomes available.

In summary, based on physical examination data, we have developed a gender-specific cascading CHD risk assessment system that utilizes a set of indicators to characterize CHD risk. This system has good performance and a wide range of application scenarios, making important contribution to the early diagnosis and treatment of CHD.

## Methods

### Study design and participants

This is a multicenter retrospective study. The data was obtained from 128 township health centers and 18 community health service centers in 7 regions under the jurisdiction of the Luzhou Municipal Health Commission from January 2018 to October 2021 (10 township health centers and 8 community health service centers in Jiangyang District; 9 township health centers and 4 community health service centers in Long Matan District; 12 township health centers and 2 community health service centers in Naxi District; 19 township health center and 1 community health service center in Lu County; 27 township health centers and 1 community health service center in Hejiang County; 25 township health centers and 1 community health service center in Xuyong County; 26 townships Health center, 1 community health service center in Gulin County). All study participants signed consent forms electronically. The data of 6 regions (Jiangyang District, Longmatan District, Lu County, Hejiang County, Xuyong County, Gulin County) were randomly divided into training data for model development and internal validation data for model examination at a ratio of 7:3. The data of one region (Naxi District) was used as external verification dataset.

### Data processing

The physical examination data was collected from the Electronic Medical Record (EMR) of Luzhou Municipal Health Commission in China from January 2018 to October 2021. These data include physical examination results of healthy persons and CHD patients. All patients with CHD have underwent coronary angiography with a diameter ≥50% of the lumen stenosis.

In order to obtain high-quality data, we preprocessed the initial data. First, we excluded features with a missing rate >50%. Then, we eliminated outliers by setting the range of feature values and then encoded text features into discrete variables. Finally, samples with missing values and duplicate samples were eliminated.

### Potential predictive variables

The physical examination data includes demographic data, vital signs, internal checkup, external checkup, laboratory data, and living habits. The demographic information and lifestyle habits were reported by individuals. The vital signs, internal checkup, and external checkup were diagnosed by professional doctors. And the laboratory data came from experimental tests and instrument diagnoses.

In order to better obtain the implicit information in these features, we integrated and processed them. First of all, based on the original features and combined with the clinical experience of doctors, some new features are calculated according to the actual needs. Evaluating a person’s obesity solely based on their height and waist circumference is not objective and rigorous. Waist-to-height ratio (WHtR) can better indicate whether a person has visceral fat accumulation. To simplify feature collection for clinical applications, we used mean systolic pressure (MSP) to replace left systolic pressure (LSP) and right systolic pressure (RSP), as well as mean diastolic pressure (MDP) to replace left diastolic pressure (LDP) and right diastolic pressure (RDP). Excessive pulse pressure difference is usually related to CHD.

The five new features were calculated as follows:

1 $${WHtR}=W/H$$

2 $${MSP}=\frac{{LSP}+{RSP}}{2}$$

3 $${MDP}=\frac{{LDP}+{RDP}}{2}$$

4 $${SPD}={LSP}-{RSP}$$

5 $${DPD}={LDP}-{RDP}$$

where *W* and *H* denote the waist circumference and height, respectively.

In the demographic information, a total of 23 symptoms were recorded (Supplementary Table 8), but not all of them were related to CHD. Therefore, we used the Chi-square test to rank features and combined incremental feature selection (IFS) strategy to select symptoms. As a result, four symptoms (Palpitation, chest tightness, dizziness, headache) were obtained, which were considered to be related to CHD. When any of the four symptoms appeared in the sample, the feature value of the symptoms was assigned as 1. When none of the four symptoms appeared, the characteristic value was set to 0. We have made a statistical description of the data after data preprocessing and feature preprocessing in Supplementary Table 1.

The questionnaire includes Smoking status (SS), Drinking frequency (DF), and Exercise frequency (EF). The characteristics of comorbidities include in the questionnaire survey are: Cerebrovascular diseases (CD), Kidney diseases (KD), Vascular diseases (VD), and Eye diseases (ED), which are discrete variables. Supplementary Table 2 and Supplementary Table 3 have shown the statistical descriptive information of the questionnaire characteristics.

### Feature selection

In model construction and analysis, a large number of information features are usually collected, as these features can provide sufficient information for models to produce good discriminative results. However, in clinical applications, we are often limited by the data collection process. The more features, the more difficult it is to collect the data. In addition, high-dimensional features can generate information redundancy or noise, which may disrupt the accuracy of predictions. Most importantly, we need to use data mining techniques, such as feature selection strategy, to identify a set of non-redundant risk factors that are closely related to the disease.

In this study, a filtering method was used to select features. The first step is to score each feature using machine learning methods or statistical methods. These scores represent the importance or significance of the feature, and then rank them based on the scores. The second step is to use IFS strategy to select the optimal set of features. The IFS strategy sequentially adds features in order of importance and forms various feature subsets. The first feature subset contains the first ranked feature, the second feature subset contains the top two features, and so on, until all feature subsets are formed. Then, an algorithm evaluates the performance of each feature subset and selects the one with the best performance as the optimal feature subset. This study used the IFS strategy combined with LR, PCC and GI sorting methods to select the optimal feature subset.

### CHD risk assessment model

Three classification models, FCN, LR, and XGBoost, were used in this paper for comparison^18–20^. These three types of algorithms have different characteristics. Among them, LR is a classical linear model. XGBoost is a tree-based model that is one of the best performing models in tree structure. FCN is an emerging multi-layer neural network model in recent years, which can learn nonlinear features. All models will use the same input variables. Since the comparison shows that FCN has the best performance, more information about FCN is introduced here. The FCN network used in this work consists of three fully connected layers, including two hidden layers and one output layer. We chose ‘Relu’ as the activation function based on experience. The gradient estimated by the ‘RMSprop’ optimizer performed gradient descent to optimize the network. To avoid overfitting, dropout was applied after each layer in the training process. The final optimized parameters have been listed in Supplementary Table 9.

### CHD risk scorecard

In order to improve the convenience of clinical application and facilitate risk stratification, we designed a gender-specific CHD risk score card^21,22^. The features used in the CHD risk score card are the same as those in CHD risk assessment model. In the scoring card, firstly, continuous risk factors were divided into 20 bins and ensured that patients with CHD and healthy persons were included in each group. Subsequently, the Chi-square test was performed on the two adjacent groups. The bins with the largest *P* value were traverses and merged until the number of bins reached the desired number. Then, the most suitable number of bins was determined (Supplementary Fig. 2 and Supplementary Fig. 3). For example, for the continuous variable MSP in Supplementary Fig. 2, the x-axis represents the number of bins (initially, each continuous variable was divided into 20 bins), and the y-axis represents the IV value. When the number of bins is 5, the IV value reaches a relatively high value. As the number of bins increases, the increase in IV value slows down. Therefore, we decided to divide the continuous variable MSP into 5 bins. Similarly, we divided Age, FBG, WHtR, PLT, BT, and BUN into 6, 5, 5, 5, 3, and 4 bins, respectively. Discrete variables do not need to be binned. For example, the Dentition variable has only two values, 1 and 0, so it is divided into two bins. The discrete variables ECG and Symptom are also divided into 2 bins each. After feature binning, we calculated the WoE of each bin and replaced WoE with the original benchmark data to ensure that all datasets could be covered by WoE.

WoE and IV were calculated as follows:

6 $${{WoE}}_{i}={\rm{ln}}\left(\frac{{HP} {\% }}{{CHD} {\% }}\right)$$

7 $${IV}=\mathop{\sum }\limits_{i=1}^{N}({HP} {\%} -{CHD} {\% })\times {{WoE}}_{i}$$

where $N$ is the number of boxes for one feature, $i$ represents each box, and *HP*% is the ratio of healthy individuals in the box to the healthy individuals in the entire feature, *CHD*% is the ratio of CHD patients in the box to the CHD patients in the entire feature.

The score in the scorecard was calculated as

8 $${Score}=A-B\times {\rm{ln}}({odds})$$

where ${odds}$ is the ratio of healthy individuals to diabetic individuals, and *A* and *B* are two constants determined as follows.

9 $${P}_{0}=A-B\times \mathrm{ln}({odds})$$

10 $${P}_{0}+{PDO}=A-B\times \mathrm{ln}(2\times {odds})$$

where ${P}_{0}$ and ${PDO}$ are the score ranges set manually, ${P}_{0}$ and ${P}_{0}+{PDO}$ (Point-to-Double Odds) correspond to two specific ratios ${odds}$ and $2\times {odds}$. $A$ and $B$ can be determined based on the two values. The CHD risk score can then be calculated in accordance with Eq. (8). In this work, the values of A and B in the male CHD risk scorecard are 41.02 and 7.64, respectively; while the values of A and B in the female CHD risk scorecard are 48.94 and 8.094, respectively.

The basic score not affected by each feature is calculated with the intercept of ln (odds). The logistic regression coefficient (Supplementary Table 10) is then considered in the calculation to determine the score of each feature in each position, as follows:

11 $${\rm{ln}}\left({odds}\right)={\theta }^{T}x={\omega }_{0}+{\omega }_{1}{x}_{1}+\ldots +{\omega }_{i}{x}_{i}+\ldots +{\omega }_{n}{x}_{n}$$

12 $$\begin{array}{l}{{score}}_{{total}}=A-B\times \left({\theta }^{T}x\right)\\ \qquad\qquad\quad=A-B\times \left({\omega }_{0}+{\omega }_{1}{x}_{1}+\ldots +{\omega }_{i}{x}_{i}+\ldots +{\omega }_{n}{x}_{n}\right)\\ \qquad\qquad\quad=\left(A-B\times {\omega }_{0}\right)-B\times {\omega }_{1}{x}_{1}-\ldots {-B\times \omega }_{i}{x}_{i}-\ldots -B\\\qquad\qquad\quad\times\, {\omega }_{n}{x}_{n}\end{array}$$

where ${\omega }_{i}$ denotes the coefficient of the *i*-th features in LR; ${\omega }_{0}$ is the intercept; and ${x}_{i}$ is the value of the *i-*th feature. The score of each feature can be multiplied by the WoE of each bin in the feature to determine the score of each bin.

The score card was calibrated to determine the total score between 0–100 points. The final scorecard consisted of the base score and the score of each bin in each feature. Finally, we divided the risk interval according to the KS curve.

### Model evaluation

A series of models were constructed, including: the feature subset model generated by the IFS strategy, the male CHD risk assessment model, the female CHD risk assessment model, the male CHD risk score card, the female CHD risk score card, and the model constructed in the symptom analysis, which needed to be evaluated^23^. In the feature selection part, the AUC value under five-fold cross validation is used to evaluate a series of feature subset models generated by the IFS strategy to select the optimal feature subset^24^. The gender specific CHD risk prediction model constructed by LR, XGBoost, and FCN was evaluated on an independent test set and external verification set by using Specificity, Recall, Accuracy, Precision, F1, AUC value and ROC curve to select the best modeling method. These indexes were also used to evaluate the performance of gender-specific CHD scoring cards on independent test sets and external validation sets. KS curve was used to compare the score distribution of CHD patients and healthy samples to set the risk interval of scorecard^25^.

### Statistical analysis

In the physical examination indicators, we counted the mean and variance of continuous variables, and the frequency of discrete variables, and calculated the *P* value of each feature between healthy people and CHD patients. Pearson’s correlation coefficient was used to measure the correlation between two features.

All statistical analyses in this study were performed using Python 3.6.

### Ethics and informed consent

This study was approved by the Ethics Committee of the University of Electronic Science and Technology of China, and the acceptance number is 1061423061626070. All study participants signed consent forms electronically.

### Reporting summary

Further information on research design is available in the Nature Research Reporting Summary linked to this article.

## Supplementary information

Supplementary materials

Reporting Summary

## Data Availability

Limited deidentified data used for the analyses presented in this work (training and testing datasets) are available to qualified researchers on request, please email the corresponding author Dr. Hui Yang at huiyang@cuit.edu.cn.

## Code availability

Limited deidentified data used for the analyses presented in this work (training and testing datasets) are available to qualified researchers on request, please email the corresponding author Dr. Hui Yang at huiyang@cuit.edu.cn.
