# Supplementary material for: A gender specific risk assessment of coronary heart disease based on physical examination data
Source: NPJ Digit Med. 2023 Jul 31;6:136. doi: 10.1038/s41746-023-00887-8 (PMC10390496; doi:10.1038/s41746-023-00887-8)
Supplement: Supplementary file 1 — Supplementary materials [file 41746_2023_887_MOESM1_ESM.pdf]

Supplementary

Supplementary Table 1. Original physical examination features

		Training cohort			External validation cohort		
Category	features	Total(n=680003)	CHD(n=39538)	Health(n=640465)	Total(n=95874)	CHD(n=5707)	Health(n=90167)
Demographics	Gender	2.0(58.2%)\	2.0(68.47%)\	2.0(57.56%)\	2.0(53.33%)\	2.0(64.48%)\	2.0(52.63%)\
		1.0(41.8%)	1.0(31.53%)	1.0(42.44%)	1.0(46.67%)	1.0(35.52%)	1.0(47.37%)
	Age	59.22±13.77	70.95±8.51	58.50±13.71	60.10±13.64	69.93±8.49	59.48±13.66
	Poverty	1.0(84.29%)\	1.0(84.64%)\	1.0(84.27%)\	1.0(92.23%)\	1.0(86.3%)\	1.0(92.61%)\
		2.0(15.71%)	2.0(15.36%)	2.0(15.73%)	2.0(7.77%)	2.0(13.7%)	2.0(7.39%)
	WHtR	0.51±0.06	0.55±0.07	0.51±0.06	0.52±0.06	0.56±0.07	0.52±0.06
	Education	2.0(49.79%)\	2.0(44.04%)\	2.0(50.15%)\	2.0(49.19%)\	2.0(48.08%)\	2.0(49.26%)\
		3.0(22.92%)\	1.0(34.73%)\	3.0(23.42%)\	1.0(23.45%)\	1.0(36.34%)\	1.0(22.63%)\
		1.0(20.38%)\	3.0(14.84%)\	1.0(19.5%)\	3.0(20.41%)\	3.0(12.18%)\	3.0(20.94%)\
		6.0(2.34%)\	16.0(1.99%)\	6.0(2.41%)\	6.0(3.18%)\	16.0(1.24%)\	6.0(3.35%)\
		16.0(1.89%)\	14.0(1.87%)\	16.0(1.88%)\	16.0(1.62%)\	14.0(0.93%)\	16.0(1.65%)\
		13.0(1.11%)\	6.0(1.23%)\	13.0(1.12%)\	13.0(0.93%)\	6.0(0.53%)\	13.0(0.96%)\
		14.0(0.94%)\	13.0(0.9%)\	14.0(0.88%)\	14.0(0.79%)\	13.0(0.46%)\	14.0(0.78%)\
		12.0(0.5%)\	12.0(0.25%)\	12.0(0.52%)\	12.0(0.33%)\	12.0(0.18%)\	12.0(0.34%)\

		15.0(0.11%)\<br>11.0(0.02%)	15.0(0.12%)\<br>11.0(0.04%)	15.0(0.11%)\<br>11.0(0.02%)	15.0(0.06%)\<br>11.0(0.03%)	15.0(0.04%)\<br>11.0(0.04%)	15.0(0.06%)\<br>11.0(0.03%)
	Occupation	8.0(51.55%)\<br>5.0(29.7%)\<br>9.0(10.78%)\<br>4.0(2.34%)\<br>2.0(2.1%)\<br>6.0(2.02%)\<br>3.0(0.76%)\<br>1.0(0.65%)\<br>7.0(0.11%)	8.0(54.21%)\<br>9.0(23.14%)\<br>5.0(10.75%)\<br>2.0(3.65%)\<br>6.0(3.04%)\<br>4.0(2.44%)\<br>3.0(1.87%)\<br>1.0(0.74%)\<br>7.0(0.16%)	8.0(51.38%)\<br>5.0(30.87%)\<br>9.0(10.01%)\<br>4.0(2.33%)\<br>2.0(2.0%)\<br>6.0(1.95%)\<br>3.0(0.69%)<br>\1.0(0.65%)\<br>7.0(0.11%)	5.0(69.91%)\<br>8.0(16.43%)\<br>9.0(7.01%)\<br>2.0(2.19%)\<br>4.0(1.9%)\<br>6.0(1.1%)\<br>3.0(0.76%)\<br>1.0(0.66%)\<br>7.0(0.05%)	5.0(61.28%)\<br>8.0(20.9%)\<br>9.0(13.05%)\<br>2.0(1.75%)\<br>4.0(0.93%)\<br>6.0(0.91%)\<br>3.0(0.7%)\<br>1.0(0.47%)	5.0(70.46%)\<br>8.0(16.15%)\<br>9.0(6.63%)\<br>2.0(2.22%)\<br>4.0(1.96%)\<br>6.0(1.11%)\<br>3.0(0.76%)\<br>1.0(0.67%)\<br>7.0(0.05%)
Vital signs	PP(Pulse pressure)	101.23±11.71	109.63±13.64	100.71±11.38	100.03±11.13	108.21±13.93	99.51±10.72
	MSP	125.80±16.14	138.72±19.18	125.00±15.59	124.18±15.38	136.55±19.57	123.39±14.73
	MDP	76.66±9.30	80.54±10.68	76.42±9.15	75.89±8.68	79.87±10.54	75.63±8.49
	BT(body tem perature)	36.52±0.25	36.49±0.25	36.52±0.25	36.58±0.25	36.56±0.26	36.58±0.25
	PF(pulse freq uency)	70.63±11.06	72.47±13.65	70.52±10.87	70.18±10.73	72.82±13.57	70.01±10.51
	HR(heart rate)	70.58±11.06	72.45±13.73	70.47±10.86	70.16±10.73	72.79±13.60	69.99±10.50

	RR(respiratory rate)	18.56±1.47	18.48±1.45	18.57±1.47	18.61±1.23	18.67±1.15	18.60±1.24
	DFAP(dorsal foot artery pulsates)	2.0(51.95%)\<br>1.0(48.01%)\<br>4.0(0.03%)\<br>8.0(0.01%)	1.0(54.43%)\<br>2.0(45.13%)\<br>4.0(0.3%)\<br>8.0(0.15%)	2.0(52.37%)\<br>1.0(47.61%)\<br>4.0(0.01%)\<br>8.0(0.01%)	2.0(74.04%)\<br>1.0(25.92%)\<br>4.0(0.02%)\<br>8.0(0.02%)	2.0(68.42%)\<br>1.0(31.14%)\<br>4.0(0.32%)\<br>8.0(0.12%)	2.0(74.4%)\<br>1.0(25.59%)\<br>8.0(0.01%)\<br>4.0(0.0%)
	Symptom	0.0(91.25%)\<br>1.0(8.75%)	0.0(84.3%)\<br>1.0(15.7%)	0.0(91.68%)\<br>1.0(8.32%)	0.0(87.82%)\<br>1.0(12.18%)	0.0(76.87%)\<br>1.0(23.13%)	0.0(88.51%)\<br>1.0(11.49%)
	RH antibody	3.0(93.65%)\<br>1.0(5.77%)<br>\2.0(0.57%)	3.0(93.26%)\<br>1.0(6.06%)\<br>2.0(0.67%)	3.0(93.68%)\<br>1.0(5.75%)\<br>2.0(0.57%)	3.0(99.03%)\<br>1.0(0.73%)\<br>2.0(0.24%)	3.0(99.51%)\<br>1.0(0.3%)\<br>2.0(0.19%)	3.0(99.0%)\<br>1.0(0.76%)\<br>2.0(0.24%)
Internal checkup<br>&<br>External checkup	AM(abdominal mass)	1.0(99.92%)\<br>2.0(0.08%)	1.0(99.93%)\<br>2.0(0.07%)	1.0(99.92%)\<br>2.0(0.08%)	1.0(99.98%)\<br>2.0(0.02%)	1.0(99.96%)\<br>2.0(0.04%)	1.0(99.98%)\<br>2.0(0.02%)
	AT(abdominal tenderness)	1.0(99.17%)<br>\2.0(0.83%)	1.0(98.35%)\<br>2.0(1.65%)	1.0(99.22%)\<br>2.0(0.78%)	1.0(98.94%)\<br>2.0(1.06%)	1.0(97.16%)\<br>2.0(2.84%)	1.0(99.05%)\<br>2.0(0.95%)
	CS(cardiac souffle)	1.0(99.92%)\<br>2.0(0.08%)	1.0(99.56%)\<br>2.0(0.44%)	1.0(99.94%)\<br>2.0(0.06%)	1.0(99.86%)\<br>2.0(0.14%)	1.0(99.46%)\<br>2.0(0.54%)	1.0(99.88%)\<br>2.0(0.12%)
	LBS(Lung breath sounds)	1.0(99.74%)\<br>2.0(0.26%)	1.0(99.04%)\<br>2.0(0.96%)	1.0(99.79%)\<br>2.0(0.21%)	1.0(99.41%)\<br>2.0(0.59%)	1.0(98.23%)\<br>2.0(1.77%)	1.0(99.49%)\<br>2.0(0.51%)
	Pharyngeal	1.0(99.8%)\	1.0(99.73%)\	1.0(99.81%)\	1.0(99.79%)\	1.0(99.7%)\	1.0(99.8%)\

	0.0(0.15%)\	0.0(0.21%)\	0.0(0.15%)\	0.0(0.13%)\	0.0(0.23%)\	0.0(0.12%)\
	2.0(0.03%)\	2.0(0.04%)\	2.0(0.03%)\	4.0(0.04%)\	2.0(0.05%)\	4.0(0.04%)\
	4.0(0.02%)	4.0(0.02%)	4.0(0.02%)	2.0(0.04%)	4.0(0.02%)	2.0(0.04%)
Vision	4.76±0.23	4.63±0.23	4.77±0.23	4.78±0.24	4.68±0.23	4.79±0.23
Dentition	0.0(54.83%)\	1.0(77.53%)\	0.0(56.83%)\	0.0(51.9%)\	1.0(76.87%)\	0.0(51.9%)\
	1.0(45.17%)	0.0(22.47%)	1.0(43.17%)	1.0(48.1%)	0.0(23.13%)	1.0(48.1%)
Hearing	1.0(98.89%)\	1.0(96.35%)\	1.0(99.05%)\	1.0(98.02%)\	1.0(96.32%)\	1.0(98.02%)\
	2.0(1.11%)	2.0(3.65%)	2.0(0.95%)	2.0(1.98%)	2.0(3.68%)	2.0(1.98%)
MF(motor function)	1.0(99.89%)\	1.0(99.69%)\	1.0(99.9%)\	1.0(99.87%)\	1.0(99.68%)\	1.0(99.87%)\
	2.0(0.11%)	2.0(0.31%)	2.0(0.1%)	2.0(0.13%)	2.0(0.32%)	2.0(0.13%)
Skin	1.0(99.09%)\	1.0(97.09%)\	1.0(99.22%)\	1.0(99.9%)\	1.0(99.72%)\	1.0(99.9%)\
	64.0(0.53%)\	64.0(2.19%)\	64.0(0.43%)\	64.0(0.04%)\	64.0(0.25%)\	64.0(0.04%)\
	32.0(0.36%)\	32.0(0.68%)\	32.0(0.34%)\	32.0(0.02%)\	8.0(0.02%)\	32.0(0.02%)\
	4.0(0.01%)\	8.0(0.02%)\	4.0(0.01%)\	4.0(0.02%)\	2.0(0.02%)	4.0(0.02%)\
	2.0(0.0%)\	4.0(0.02%)\	2.0(0.0%)\	2.0(0.01%)\		2.0(0.01%)\
	8.0(0.0%)\	2.0(0.01%)	16.0(0.0%)\	8.0(0.01%)		8.0(0.01%)
	16.0(0.0%)		8.0(0.0%)			
Sclera	1.0(99.99%)\	1.0(99.97%)\	1.0(99.99%)\	1.0(99.98%)\	1.0(99.95%)\	1.0(99.98%)\
	2.0(0.0%)\	4.0(0.01%)\	2.0(0.0%)\	2.0(0.01%)\	2.0(0.04%)\	2.0(0.01%)\
	8.0(0.0%)\	8.0(0.01%)\	8.0(0.0%)\	4.0(0.01%)	4.0(0.02%)	4.0(0.01%)

	4.0(0.0%)	2.0(0.01%)	4.0(0.0%)			
LG(lymph gland)	1.0(99.94%)\	1.0(99.96%)\	1.0(99.94%)\	1.0(99.98%)\	1.0(99.98%)\	1.0(99.98%)\
	2.0(0.03%)\	8.0(0.02%)\	2.0(0.04%)\	8.0(0.01%)\	2.0(0.02%)	8.0(0.01%)\
	8.0(0.02%)\	2.0(0.01%)\	8.0(0.02%)\	2.0(0.01%)\		2.0(0.01%)\
	4.0(0.0%)	4.0(0.0%)	4.0(0.0%)	4.0(0.0%)		4.0(0.0%)
PBC(Pulmonary barrel chest)	1.0(99.81%)\	1.0(98.75%)\	1.0(99.88%)\	1.0(99.58%)\	1.0(98.04%)\	1.0(99.58%)\
	2.0(0.19%)	2.0(1.25%)	2.0(0.12%)	2.0(0.42%)	2.0(1.96%)	2.0(0.42%)
LR(Lung rale)	1.0(99.86%)\	1.0(99.43%)\	1.0(99.89%)\	1.0(99.82%)\	1.0(99.3%)\	1.0(99.82%)\
	2.0(0.06%)\	4.0(0.3%)\	2.0(0.05%)\	2.0(0.11%)\	4.0(0.33%)\	2.0(0.11%)\
	4.0(0.06%)\	2.0(0.2%)\	4.0(0.04%)\	4.0(0.05%)\	2.0(0.3%)\	4.0(0.05%)\
	8.0(0.01%)	8.0(0.07%)	8.0(0.01%)	8.0(0.01%)	8.0(0.07%)	8.0(0.01%)
RH(rhythm of the heart)	1.0(95.87%)\	1.0(92.41%)\	1.0(96.09%)\	1.0(97.4%)\	1.0(93.99%)\	1.0(97.4%)\
	2.0(3.98%)\	2.0(6.41%)\	2.0(3.83%)\	2.0(2.49%)\	2.0(5.33%)\	2.0(2.49%)\
	4.0(0.14%)	4.0(1.18%)	4.0(0.08%)	4.0(0.11%)	4.0(0.68%)	4.0(0.11%)
ELE(edema of lower extremity)	1.0(99.85%)\	1.0(98.78%)\	1.0(99.91%)\	1.0(99.51%)\	1.0(97.98%)\	1.0(99.51%)\
	8.0(0.1%)\	8.0(0.98%)\	8.0(0.04%)\	8.0(0.25%)\	8.0(1.42%)\	8.0(0.25%)\
	2.0(0.04%)\	2.0(0.17%)\	2.0(0.03%)\	4.0(0.12%)\	4.0(0.4%)\	4.0(0.12%)\
	4.0(0.02%)	4.0(0.07%)	4.0(0.01%)	2.0(0.12%)	2.0(0.19%)	2.0(0.12%)
AD(anus dre)	1.0(99.99%)\	1.0(99.98%)\	1.0(99.99%)\	1.0(100.0%)\	1.0(100.0%)	1.0(100.0%)\
	16.0(0.0%)\	16.0(0.01%)\	16.0(0.0%)\	16.0(0.0%)		16.0(0.0%)

		2.0(0.0%)\	2.0(0.01%)\	2.0(0.0%)\			
		4.0(0.0%)\	4.0(0.0%)	4.0(0.0%)\			
		8.0(0.0%)		8.0(0.0%)			
Laboratory values	FBG(fasting blood-glucose)	5.39±1.31	6.21±2.40	5.34±1.20	5.41±1.39	6.17±2.58	5.36±1.26
	Hemoglobin	133.76±17.01	132.04±15.63	133.86±17.09	134.36±15.79	132.43±15.10	134.48±15.83
	Hemameba	6.13±2.05	6.25±2.01	6.13±2.06	5.99±1.78	6.21±1.85	5.97±1.78
	PLT(Platelet count)	209.43±63.81	200.04±63.98	210.01±63.76	206.64±56.64	203.59±58.51	206.83±56.52
	PRO(protein)	0.0(93.87%)\	0.0(87.98%)\	0.0(94.23%)\	0.0(94.65%)\	0.0(88.82%)\	0.0(94.65%)\
		1.0(5.16%)\	1.0(9.43%)\	1.0(4.9%)\	1.0(4.37%)\	1.0(8.73%)\	1.0(4.37%)\
		2.0(0.73%)\	2.0(2.12%)\	2.0(0.64%)\	2.0(0.55%)\	2.0(1.45%)\	2.0(0.55%)\
		3.0(0.21%)\	3.0(0.43%)\	3.0(0.2%)\	3.0(0.42%)\	3.0(1.0%)	3.0(0.42%)\
		4.0(0.03%)	4.0(0.03%)	4.0(0.03%)	4.0(0.01%)		4.0(0.01%)
	UGLU (urine glucose)	0.0(97.61%)\	0.0(94.83%)\	0.0(97.78%)\	0.0(98.74%)\	0.0(95.64%)\	0.0(98.74%)\
		1.0(1.75%)\	1.0(2.78%)\	1.0(1.69%)\	1.0(0.72%)\	1.0(1.59%)\	1.0(0.72%)\
		3.0(0.3%)\	3.0(1.47%)\	2.0(0.25%)\	3.0(0.27%)\	3.0(1.58%)\	3.0(0.27%)\
		2.0(0.28%)\	2.0(0.69%)\	3.0(0.23%)\	2.0(0.2%)\	2.0(0.86%)\	2.0(0.2%)\
		4.0(0.06%)	4.0(0.23%)	4.0(0.05%)	4.0(0.07%)	4.0(0.33%)	4.0(0.07%)

UAB(urine acetone bodies)	0.0(97.61%)\<br>1.0(1.84%)\<br>2.0(0.37%)\<br>3.0(0.14%)\<br>4.0(0.04%)	0.0(96.96%)\<br>1.0(2.47%)\<br>2.0(0.34%)\<br>3.0(0.16%)\<br>4.0(0.07%)	0.0(97.65%)\<br>1.0(1.81%)\<br>2.0(0.37%)\<br>3.0(0.13%)\<br>4.0(0.04%)	0.0(97.81%)\<br>1.0(1.74%)\<br>2.0(0.33%)\<br>3.0(0.1%)\<br>4.0(0.01%)	0.0(97.49%)\<br>1.0(2.03%)\<br>2.0(0.28%)\<br>3.0(0.19%)	0.0(97.81%)\<br>1.0(1.74%)\<br>2.0(0.33%)\<br>3.0(0.1%)\<br>4.0(0.01%)
UOB(urine occult blood)	0.0(88.84%)\<br>1.0(7.56%)\<br>2.0(2.07%)\<br>3.0(1.5%)\<br>4.0(0.04%)	0.0(83.94%)\<br>1.0(11.5%)\<br>2.0(2.9%)\<br>3.0(1.66%)\<br>4.0(0.01%)	0.0(89.15%)\<br>1.0(7.31%)\<br>2.0(2.02%)\<br>3.0(1.49%)\<br>4.0(0.04%)	0.0(89.65%)\<br>1.0(6.4%)\<br>2.0(2.23%)\<br>3.0(1.69%)\<br>4.0(0.03%)	0.0(86.3%)\<br>1.0(8.83%)\<br>2.0(2.77%)\<br>3.0(2.1%)	0.0(89.65%)\<br>1.0(6.4%)\<br>2.0(2.23%)\<br>3.0(1.69%)\<br>4.0(0.03%)
OBS(occult blood in stool)	0.0(99.96%)\<br>1.0(0.04%)	0.0(99.99%)\<br>1.0(0.01%)	0.0(99.96%)\<br>1.0(0.04%)	0.0(99.99%)\<br>1.0(0.01%)	0.0(100.0%)	0.0(99.99%)\<br>1.0(0.01%)
SGPT(serum glutamic pyruvic transaminase)	22.73±12.41	22.54±12.06	22.74±12.43	23.37±12.77	23.05±12.64	23.39±12.78
SGOT(serum glutamic oxalacetic transaminase)	25.68±10.28	25.39±9.83	25.70±10.30	25.38±9.48	25.44±9.39	25.37±9.49
TBil(total bilirubin)	12.36±5.93	12.66±5.80	12.34±5.93	11.83±5.56	12.09±5.50	11.82±5.56

	SC(serum cre atinine)	74.34±18.98	76.41±20.22	74.21±18.89	73.18±17.60	74.91±19.79	73.07±17.45
	BUN(blood ur ea nitrogen)	5.57±2.61	5.88±2.42	5.55±2.62	5.75±2.31	5.99±2.17	5.73±2.32
	TC(total chole sterol)	4.93±1.37	5.08±1.28	4.92±1.38	5.05±1.14	5.22±1.17	5.04±1.14
	Triglyceride	1.69±1.52	1.82±1.58	1.69±1.51	1.64±1.48	1.87±1.60	1.63±1.47
	SLDLC(Serum low density lipoprotein cholesterol)	2.64±1.00	2.75±1.08	2.64±0.99	2.95±0.94	3.05±1.04	2.95±0.93
	SHDLC(Serum high density lipoprotein cholesterol)	1.61±0.86	1.56±0.72	1.61±0.87	1.63±0.64	1.56±0.59	1.64±0.64
	HBsAg	0.0(99.88%)\<br>1.0(0.12%)	0.0(99.97%)\<br>1.0(0.03%)	0.0(99.88%)\<br>1.0(0.12%)	0.0(99.98%)\<br>1.0(0.02%)	0.0(100.0%)	0.0(99.98%)\<br>1.0(0.02%)
	ECG	0.0(53.8%)\<br>1.0(46.2%)	1.0(67.31%)\<br>0.0(32.69%)	0.0(55.1%)\<br>1.0(44.9%)	0.0(52.39%)\<br>1.0(47.61%)	1.0(74.26%)\<br>0.0(25.74%)	0.0(52.39%)\<br>1.0(47.61%)

Gender: 2.0(female)\1.0(male)

AM(abdominal mass): 1.0(normal)\2.0(abnormal)

AT(abdominal tenderness): 1.0(normal)\2.0(abnormal)

CS(cardiac souffle): 1.0(normal)\2.0(abnormal)

LBS(lung breath sounds): 1.0(normal)\2.0(abnormal)

ECG: 0.0(normal)\1.0(abnormal)

Poverty: 1.0(no)\2.0(yes)

RHA (RH antibody): 3.0(unknown)\1.0(positive)\2.0(negative)

Education: 2.0(primary school)\3.0(junior middle school)\1.0(Illiterate and semi-literate)\6.0(unknown)\16.0(high school)\13.0(Colleges and colleges)\14.0(Specialized secondary schools)\12.0(Bachelor's degree)\15.0(Technical school)\11.0(Graduate students)

Occupation: 8.0(Other practitioners who are not suitable for classification)\5.0(Agricultural production personnel)\9.0(No professional personnel)\4.0(Service personnel)\2.0(Professional and technical personnel)\6.0(Production and transportation personnel)\3.0(Administrative personnel)\1.0(Personnel of state organs)\7.0(soldiers)

DFAP(dorsal foot artery pulsates): 2.0(touch bilateral symmetry)\1.0(untouched)\4.0(touch the left side weak or disappear)\8.0(touch the right side weak or disappear)

Hearing: 1.0(normal)\2.0(abnormal)

MF(motor function):1.0(normal)\2.0(abnormal)

Skin: 1.0(normal)\64.0(other)\32.0(Pigmentation)\4.0(pale)\2.0(hot flashes)\8.0(cyanosis)\16.0(yellow dye)

Sclera: 1.0(normal)\2.0(yellow dye)\8.0(other)\4.0(congestion)

LG(lymph gland): 1.0(untouched)\2.0(Supraclavicular)\4.0(axillary)\8.0(other)

PBC(Pulmonary barrel chest): 1.0(normal)\2.0(abnormal)`

LR(Lung rale): 1.0(normal)\2.0(dry rales)\4.0(Wet rales)\8.0(other)

RH(rhythm of the heart): 1.0(normal)\2.0(arrhythmia)\4.0(absolutely irregular heart rate)

ELE(edema of lower extremity): 1.0(normal)\8.0(bilateral symmetry)\2.0(unilateral)\4.0(bilateral asymmetry)

AD(anus dre): 1.0(normal)\16.0(other)\2.0(tenderness)\4.0(bag piece)\8.0(Prostate abnormalities)

PRO(protein): 0.0(-)\1.0(1+)\2.0(2+)\3.0(3+)\4.0(4+)

UGLU (urine glucose): 0.0(-)\1.0(1+)\2.0(2+)\3.0(3+)\4.0(4+)

UAB(urine acetone bodies): 0.0(-)\1.0(1+)\2.0(2+)\3.0(3+)\4.0(4+)

UOB(urine occult blood): 0.0(-)\1.0(1+)\2.0(2+)\3.0(3+)\4.0(4+)

OBS(occult blood in stool): 0.0(normal)\1.0(abnormal)

HBsAg: 0.0(normal)\1.0(abnormal)

Symptom: 0.0(normal)\1.0(Palpitation, chest tightness, dizziness, headache)

DH(dietary habits): 0.0(normal)\1.0(like Salt, oil, sugar)

Dentition： 0.0(normal)\1.0(Dental absence, caries and dentures)

Supplementary Table 2. Statistical description of lifestyle habits

features	Total(n=775590)	CHD(n=45242)	Health(n=730348)
ST	1.0(72.29%)\	1.0(80.27%)\	1.0(71.79%)\
	2.0(2.27%)\	2.0(6.8%)\	2.0(1.2%)\
	3.0(25.43%)	3.0(12.89%)	3.0(26.21%)
DF	1.0(77.66%)\	1.0(83.64%)\	1.0(77.29%)\
	2.0(13.03%)\	2.0(10.57%)\	2.0(13.18%)\
	3.0(9.21%)	3.0(5.78%)	3.0(9.42%)
EF	1.0(8.58%)\	1.0(20.14%)\	1.0(7.9%)\
	2.0(2.75%)\	2.0(4.9%)\	2.0(2.6%)\
	3.0(2.15%)\	3.0(3.5%)\	3.0(2.1%)\
	4.0(86.52%)	4.0(71.35%)	4.0(87.45%)

ST(smoking status): 1.0(Never smoked)\3.0(smoking)\2.0(smoking cessation)

DF(drinking frequency): 1.0(never)\2.0(occasionally)\3.0 (often)

EF(exercise frequency): 4.0(Don't exercise)\1.0(each day)\2.0(More than once a week)\3.0(once in a while)

Supplementary Table 3. Statistical description of comorbidities

features	Total(n=828884)	CHD(n=47947)	Health(n=780938)
CD	1.0(99.62%)\ 2.0(0.38%)	1.0(94.92%)\ 2.0(5.08%)	1.0(99.91%)\ 2.0(0.09%)
KD	1.0(95.33%)\ 2.0(4.67%)	1.0(80.16%)\ 2.0(19.84%)	1.0(96.26%)\ 2.0(3.74%)
VD	1.0(99.97%)\ 2.0(0.03%)	1.0(99.67%)\ 2.0(0.33%)	1.0(99.99%)\ 2.0(0.01%)
ED	1.0(99.50%)\ 2.0(0.50%)	1.0(98.15%)\ 2.0(1.85%)	1.0(99.58%)\ 2.0(0.42%)

CD (Cerebrovascular diseases): 1.0(no)\2.0(yes)

KD (Kidney diseases): 1.0(no)\2.0(yes)

VD (Vascular diseases): 1.0(no)\2.0(yes)

ED (Eye diseases): 1.0(no)\2.0(yes)

Supplementary Table 4. Feature selection step 1: Single feature AUC and AUC under IFS strategy.

	Features	Features_AUC	IFS_5V_AUC
1	Age	0.778286196	0.778286196
2	MSP	0.715961006	0.809631782
3	WHtR	0.704078943	0.835286674
4	PP	0.696911738	0.834569373
5	Dentition	0.671804773	0.863496387
6	Vision	0.669264054	0.863939968
7	FBG	0.626975536	0.870013451
8	MDP	0.61703819	0.870013376
9	ECG	0.612073987	0.873955609
10	Gender	0.554536874	0.875457492
11	BUN	0.554059241	0.875514288
12	PLT	0.550684515	0.876021052
13	Triglyceride	0.547785148	0.875842338
14	TC	0.539414541	0.874853324
15	Symptom	0.536911335	0.876272366
16	BT	0.535221271	0.876577335
**17**	**Hemoglobin**	**0.535015801**	**0.876585884**
18	DFAP	0.53302596	0.873471973
19	SLDLC	0.531494701	0.873055903
20	PRO	0.531493057	0.873506233
21	HR	0.531273521	0.873620037
22	PF	0.531261359	0.873611686
23	SHDLC	0.525894773	0.873739601
24	UOB	0.525543511	0.873784689
25	Hemameba	0.522368555	0.873671521
26	RR	0.521350455	0.87394909
27	SC	0.52081514	0.873955998
28	RH	0.518643484	0.874505856
29	DH	0.518473374	0.875548524
30	TBil	0.518369333	0.875556639
31	UGLU	0.514922387	0.875576937
32	Hearing	0.513511939	0.875690961
33	Skin	0.51066118	0.876316913

34	SGOT	0.508833463	0.876441268
35	ELE	0.505678073	0.876890197
36	PBC	0.505625018	0.877593437
37	AT	0.504312824	0.877695609
38	LBS	0.503711487	0.877763438
39	UAB	0.503393203	0.877704304
40	SGPT	0.502307701	0.878007455
41	LR	0.502292918	0.878047104
42	CS	0.501903079	0.878196336
43	MF	0.501072213	0.878185997
44	HBsAg	0.500478416	0.878179715
45	Pharyngeal	0.500233861	0.87818843
46	Sclera	0.500122242	0.878195907
47	OBS	0.500120229	0.878190179
48	AD	0.500060414	0.878189793
49	LG	0.499927562	0.878189792
50	AM	0.499677916	0.878152428
51	RHA	0.483234175	0.876503746

Supplementary Table 5. Feature selection step 3: GINI impurity and AUC in IFS strategy.

	Features	GINI impurity	IFS_5V_AUC
1	Dentition	0.35149533	0.67180477
2	Age	0.29890698	0.83079147
3	MSP	0.062131204	0.85495388
4	WHtR	0.061418686	0.87299490
5	ECG	0.060486	0.87647083
6	Symptom	0.045007918	0.87790954
7	Gender	0.040363356	0.87971195
8	FBG	0.026229462	0.88346403
9	BT	0.011819883	0.88373019
10	PLT	0.01094614	0.88441608
**11**	**BUN**	**0.010596671**	**0.88508630**
12	TC	0.010409742	0.88508349
13	Triglyceride	0.009188587	0.88463225

Supplementary Table 6. Univariate analysis for the physical examination features in training dataset

Category	Features	Male				Female			
		Total (n=284257)	CHD (n=12466)	Health (n=271791)	P-value	Total (n=395746)	CHD (n=27072)	Health (n=368674)	P-value
Demographics	Age	60.15±13.86	71.44±8.33	59.63±13.84	<0.001	58.56±13.67	70.72±8.59	57.67±13.55	<0.001
	WHtR	0.49±0.05	0.53±0.06	0.49±0.05	<0.001	0.52±0.06	0.56±0.07	0.52±0.06	<0.001
Vital signs	MSP	127.41±15.76	138.01±18.75	126.92±15.43	<0.001	124.64±16.31	139.04±19.37	123.59±15.55	<0.001
	Symptom	0.0(91.27%)\1.0(8.73%)	0.0(84.69%)\1.0(15.31%)	0.0(91.57%)\1.0(8.43%)	<0.001	0.0(91.24%)\1.0(8.76%)	0.0(84.12%)\1.0(15.88%)	0.0(91.76%)\1.0(8.24%)	<0.001
	BT	36.53±0.25	36.50±0.24	36.53±0.25	<0.001	36.51±0.25	36.48±0.25	36.51±0.25	<0.001
External checkup	Dentition	0.0(55.71%)\1.0(44.29%)	0.0(23.01%)\1.0(76.99%)	0.0(57.21%)\1.0(42.79%)	<0.001	0.0(54.21%)\1.0(45.79%)	1.0(77.78%)\0.0(22.22%)	0.0(56.55%)\1.0(43.45%)	<0.001
Laboratory values	FBG	5.40±1.35	6.14±2.34	5.37±1.27	<0.001	5.39±1.29	6.25±2.43	5.33±1.14	<0.001
	PLT	205.14±62.17	189.41±60.9	205.86±62.13	<0.001	212.52±64.79	204.93±64.75	213.08±64.76	<0.001
	BUN	5.80±2.68	6.10±2.57	5.79±2.68	<0.001	5.41±2.54	5.79±2.34	5.38±2.55	<0.001
	ECG	1.0(50.57%)\2.0(49.43%)	1.0(29.1%)\2.0(70.9%)	1.0(51.56%)\2.0(48.44%)	<0.001	1.0(56.12%)\2.0(43.88%)	2.0(65.65%)\1.0(34.35%)	1.0(57.72%)\2.0(42.28%)	<0.001

Supplementary Table 7. Univariate analysis for the physical examination features in external dataset

Category	Features	Male				Female			
		Total (n=)	CHD (n=2027)	Health (n=42715)	P-value	Total (n=)	CHD (n=3680)	Health (n=47452)	P-value
Demographics	Age	61.01±13.59	70.24±8.35	60.57±13.63	<0.001	59.31±13.63	69.76±8.56	58.50±13.62	<0.001
	WHtR	0.51±0.05	0.53±0.06	0.50±0.05	<0.001	0.53±0.06	0.57±0.07	0.53±0.06	<0.01
Vital signs	MSP	125.47±14.65	136.28±19.02	124.95±14.21	<0.001	123.04±15.90	136.70±19.86	121.99±15.04	<0.001
	Symptom	0.0(88.08%)\1.0 (11.92%)	0.0(88.62%)\1 .0(11.38%)	0.0(76.62%)\1 .0(23.38%)	<0.001	0.0(87.59%)\1 .0(12.41%)	0.0(88.42%)\1 .0(11.58%)	0.0(77.01%)\1 .0(22.99%)	<0.001
	BT	36.58±0.25	36.57±0.27	36.59±0.25	<0.001	36.58±0.25	36.55±0.25	36.58±0.25	<0.001
External checkup	Dentition	1.0(52.85%)\0.0 (47.15%)	1.0(51.68%)\0 .0(48.32%)	1.0(77.55%)\0 .0(22.45%)	<0.001	1.0(53.93%)\0 .0(46.07%)	1.0(52.11%)\0 .0(47.89%)	1.0(77.39%)\0 .0(22.61%)	<0.001
Laboratory values	FBG	5.43±1.39	6.01±2.40	5.40±1.32	<0.001	5.39±1.39	6.26±2.67	5.32±1.21	<0.001
	PLT	202.14±55.61	197.87±58.17	202.34±55.48	<0.001	210.57±57.25	206.74±58.46	210.87±57.14	<0.001
	BUN	6.01±2.35	6.17±2.38	6.00±2.35	<0.001	5.52±2.25	5.89±2.05	5.49±2.26	<0.001
	ECG	2.0(52.43%)\1.0 (47.57%)	2.0(51.2%)\1. 0(48.8%)	2.0(78.24%)\1 .0(21.76%)	<0.001	1.0(53.63%)\2 .0(46.37%)	1.0(55.62%)\2 .0(44.38%	2.0(72.07%)\1 .0(27.93%)	<0.001

Supplementary Table 8. IFS results for symptom characteristics

Features	IFS_AUC
**Headache**	**0.6222**
**Dizzy**	**0.6231**
**Heart palpitations.**	**0.6253**
**Chest distress**	**0.6264**
Chest pain	0.6235
Chronic cough	0.6233
Coughing up phlegm	0.6291
Dyspnea	0.6292
Drink more	0.6295
Urine more	0.6294
Weight loss	0.6305
Fatigue	0.6304
Pain in the joints	0.6340
Blurred vision	0.6347
Hands and feet numbness	0.6348
Urgency	0.6350
The urine pain	0.6351
Constipation	0.6351
Diarrhea	0.6352
Nausea and vomiting	0.6358
Blurred vision	0.6370
Tinnitus	0.6361
Breast pain	0.6361

Supplementary Table 9. Each model parameter in the CHD risk assessment model and scorecard

CHD risk assessment model			CHD risk score card
FCN	LR	XGBOOST	LR
'layer1_in_features': 10	‘C': 1	‘base_score': 0.5	‘C': 1
'layer1_out_features': 7	'class_weight': 0: 1, 1: 16	'booster': 'gbtree'	'class_weight': 0: 1, 1: 16
'layer1_bias': True	'dual': False	'colsample_bylevel': 1	'dual': False
'layer1_forward_function': Relu	'fit_intercept': True	'colsample_bynode': 1	'fit_intercept': True
'layer2_in_features': 7	'intercept_scaling': 1	'colsample_bytree': 1	'intercept_scaling': 1
'layer2_ou_features': 4	'l1_ratio': None	'gamma': 0	'l1_ratio': None
'layer2_bias': True	'max_iter': 1000	'learning_rate': 0.00100000005	'max_iter': 1000
'layer2_forward_function': Relu	'multi_class': 'auto'	'max_delta_step': 0	'multi_class': 'auto'
'layer3_in_features': 4	'n_jobs': None	'max_depth': 6	'n_jobs': None
'layer3_out_features': 2	'penalty': 'l1'	'min_child_weight': 1	'penalty': 'l1'
'layer3_bias': True	'random_state': 10	'missing': None	'random_state': 10
'layer3_forward_function': Softmax	'solver': 'liblinear'	'n_estimators': 200	'solver': 'liblinear'
'layer2_bias': True	'tol': 0.0001	'n_jobs': 8	'tol': 0.0001
'layer4_in_features': 2	'verbose': 0	'nthread': None	'verbose': 0
'layer4_out_features': 1	'warm_start': False	'objective': 'binary:logistic'	'warm_start': False
'layer4_bias': True		'random_state': 15	LR
'layer4_forward_function': Softmax		'reg_alpha': 0	‘C': 1
		'reg_lambda': 1	'class_weight': 0: 1, 1: 16
		'scale_pos_weight': 12	'dual': False
		'seed': None	
		'silent': None	
		'subsample': 1	
		'verbosity': None	
		'eta': 0.01	

Supplementary Table 10. Coefficients of CHD risk scorecard models established by logistic regression

	Features	Male	Female
1	FBG	-0.66433192	-0.69315899
2	Age	-0.82118255	-0.80446091
3	MSP	-0.60222084	-0.59610749
4	WHtR	-0.7864424	-0.63151727
5	PLT	-0.52356847	-0.56922886
6	BT	-0.66301309	-0.76229746
7	BUN	-0.5267144	-0.35161862
8	Dentition	-0.94089105	-0.98428877
9	ECG	-0.56541145	-0.67876396
10	Symptom	0.90768298	-0.89953396
11	lr.intercept	-0.31115537	0.11055879

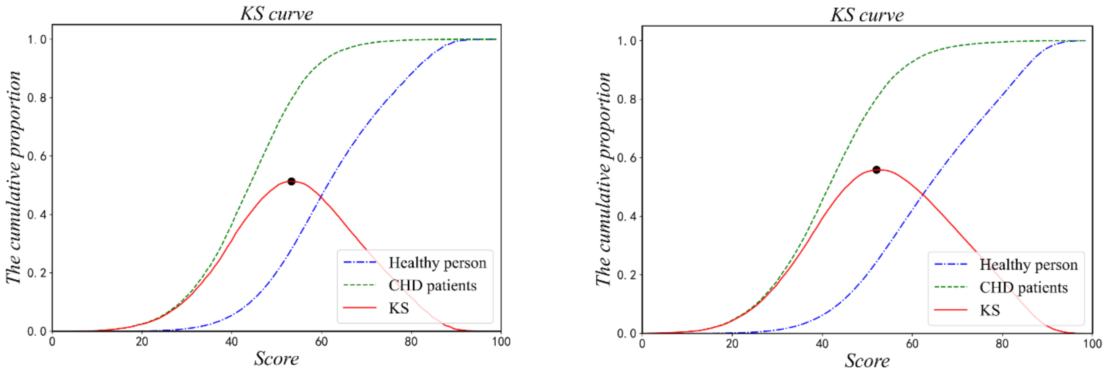

(a) (b)

Supplementary Fig. 1: KS curve for CHD risk scorecard: (a) KS curve for male, (b) KS curve for female.

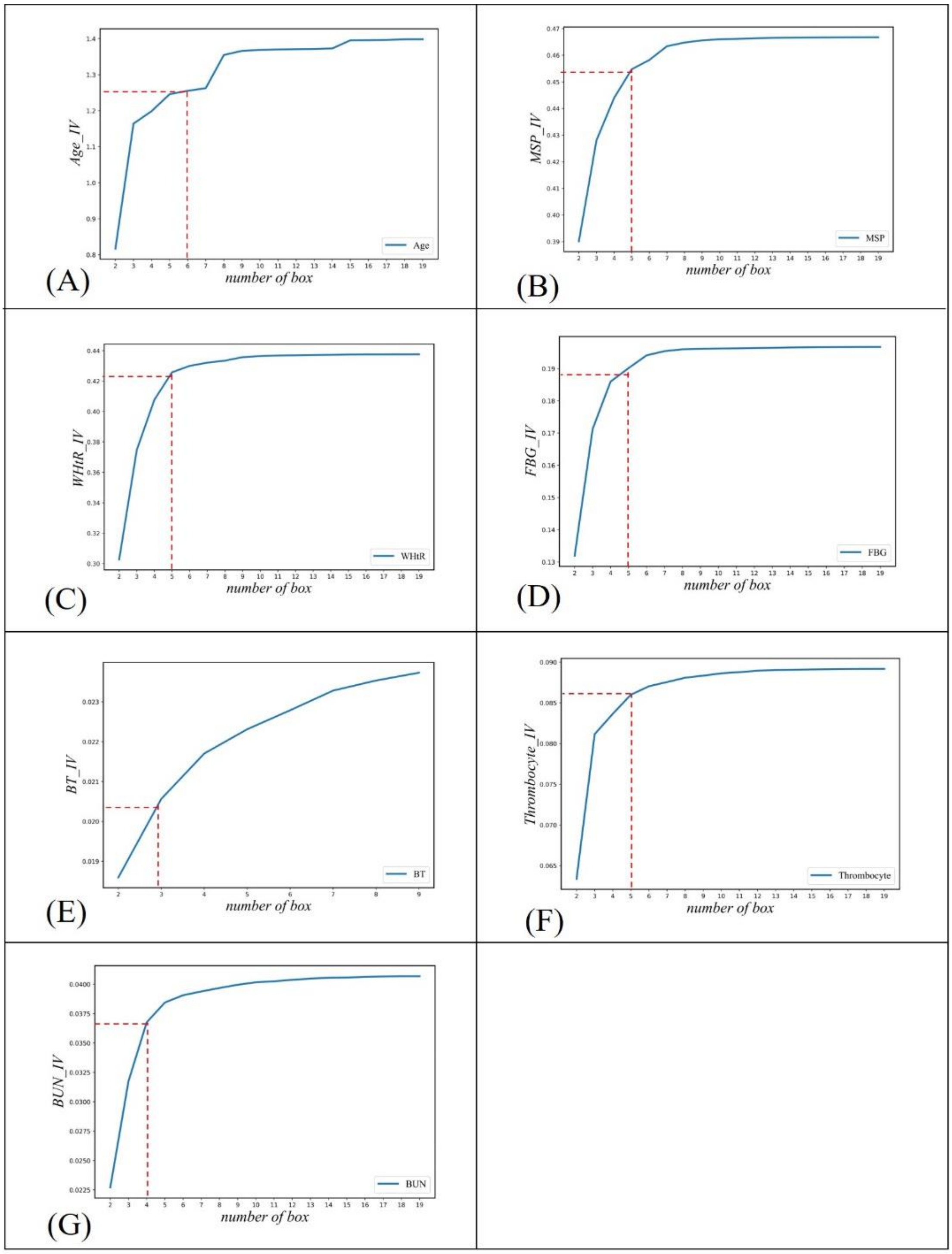

Supplementary Fig. 2: IV values for male continuous features under each bin

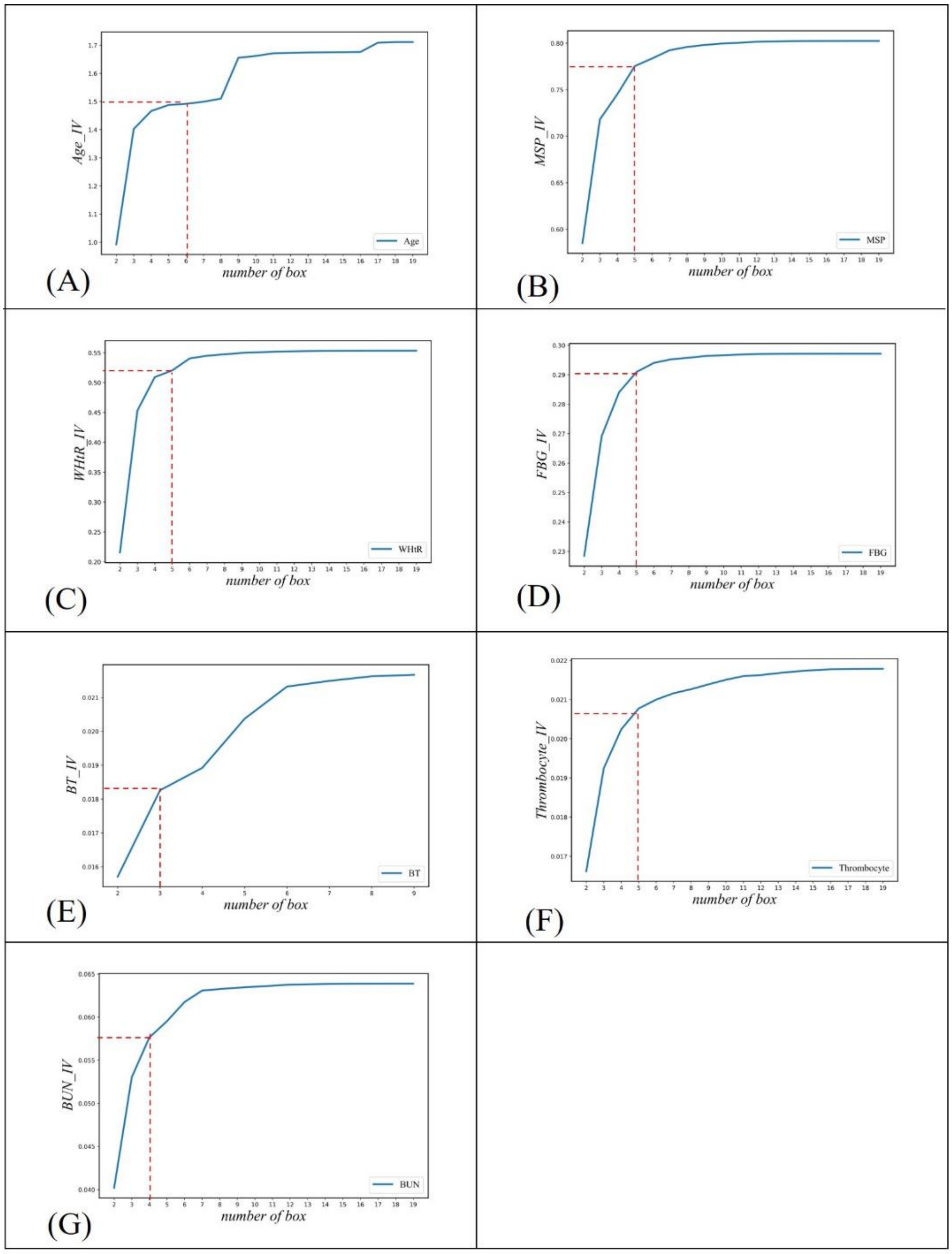

Supplementary Fig. 3: IV values for female continuous features under each bin.
